# Overuse of a molecular infectious diarrhea panel in acute care: an opportunity for diagnostic stewardship

**DOI:** 10.1017/ash.2026.10753

**Published:** 2026-06-23

**Authors:** Bushra Alhothli, Michael Payne, Aleksandra Stefanovic, Victor Leung, Christopher F. Lowe, Patrick Tang, Marc G. Romney, Nancy Matic

**Affiliations:** 1 Department of Pathology and Laboratory Medicine, University of British Columbia, Vancouver, Canada; 2 Division of Medical Microbiology and Virology, St. Paul’s Hospital, Vancouver, Canada

## Abstract

A retrospective review of infectious diarrhea panel (IDP) utilization identified 62% of orders were non-adherent to clinical guidelines. Guideline-discordant orders were significantly less likely to (1) test positive for any target or (2) have impact on patient care. Improving IDP ordering for value-based healthcare is an important diagnostic stewardship intervention.

## Background

Acute infectious diarrhea remains a major cause of morbidity worldwide, with significant healthcare and economic burden.^1^ Traditional stool diagnostic methods (bacterial culture, antigen assays, and microscopy) are limited by long turnaround time and low sensitivity.^2^ Over the past decade, multiplex molecular infectious diarrhea panels (IDP) have emerged as rapid, sensitive diagnostic tools capable of detecting 20–30 bacterial, viral, and parasitic targets within hours.^3^ Clinical studies demonstrate IDP improve diagnostic yield, expedite targeted therapy, and may reduce length of hospital stay and unnecessary antimicrobial use.^4,5^

However, IDP benefits are counterbalanced by concerns over cost, appropriateness of use, and diagnostic stewardship. Recognizing these challenges, guidelines from the Infectious Diseases Society of America (IDSA) and the Guidelines and Protocol Advisory Committee (GPAC) of British Columbia emphasize limiting IDP to patients with severe or persistent symptoms and avoiding repeat testing within a single diarrheal case.^1,6^

In October 2023, our hospital-based clinical microbiology laboratory introduced an IDP (BioFire® FilmArray® Gastrointestinal [GI] Panel, bioMérieux) to replace traditional bacterial stool culture and ova and parasite (O&P) examination for intestinal protozoa. We reviewed IDP utilization for appropriateness (adherence to clinical guidelines) and impact on patient management.

## Methods

We conducted a retrospective review of IDP orders received in the laboratory between October 2023 and July 2025. Orders were submitted from the tertiary care hospital (St. Paul’s Hospital) and one community acute care hospital (Mount Saint Joseph Hospital) in Vancouver, British Columbia including Emergency Departments (ED), inpatient wards, and nearby outpatient clinics. A subset of 25–30 orders were reviewed each quarter (January, April, July, and November of each year) selected randomly by alphabetical order of patient name. Classification as an appropriate “guideline-concordant” order was based on criteria established by GPAC (new diarrhea [≥3 loose stool in a 24-hour period] without an alternate explanation; AND diarrhea persisting >7 days, OR presenting with severe symptoms such as bloody stool, fever, severe pain, OR patient is severely immunocompromised).^6^ IDP results were further analyzed for rejection rate (samples are routinely rejected by the laboratory for tests repeated within 7 days, inpatients admitted >72 hours, or inappropriate container/labeling), pathogen detection, and clinical impact on patient management. Statistical analysis (Fisher’s exact test) was performed using RStudio(1.4.1717).

## Results

**Figure 1.**
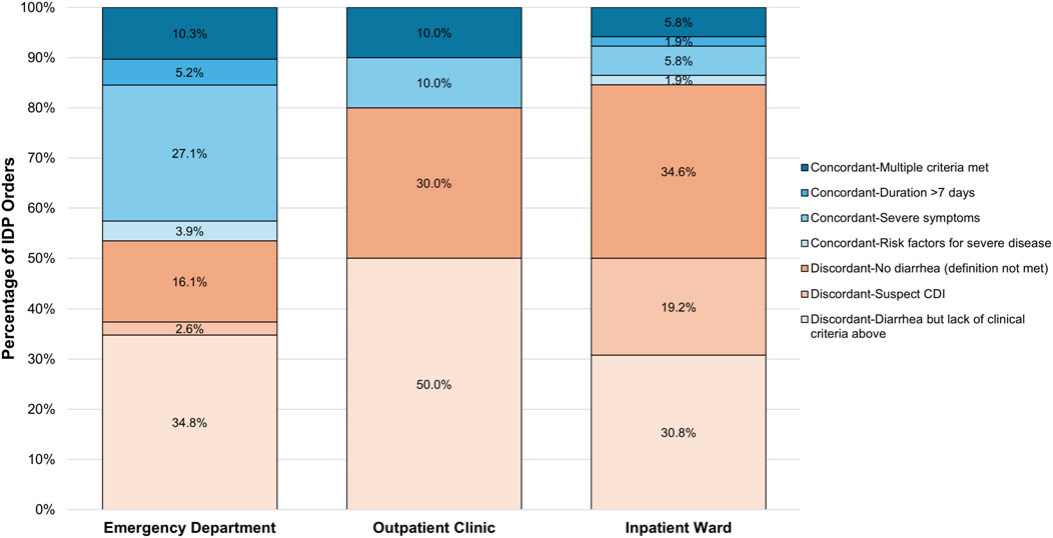
Figure 1 long description. IDP (Infectious diarrhea panel) requests by ordering location and concordance with the British Columbia GPAC (Guidelines and protocol advisory committee) criteria.

A total of 4,631 stool samples for IDP testing were received in the laboratory during this study’s time frame. A random subset of 233 orders were reviewed; sixteen (16) outpatient samples were excluded from the study due to lack of available clinical documentation. Of the remaining 217 orders which underwent review, only 38% (82/217) were determined to be guideline-concordant. The rate of guideline-concordant IDP orders did not change over time but did vary by ordering location (46% [72/155] from ED, 20% [2/10] from outpatient clinics, and 15% [8/52] from inpatient wards), where orders from ED were five times more likely to be concordant compared to the other sites (Odds Ratio [OR] = 5.9, 95% Confidence Interval [CI] = 2.7, 14.1). Orders were placed by a medical doctor in 95% (78/82) of concordant cases versus 82% (111/135) of discordant cases, with the remainder of orders being placed by nursing staff. Notably, for a substantial proportion of orders (21% [46/217]), patients did not meet definition for diarrheal illness (Figure 1).

Guideline-concordant IDP orders were three times more likely to test positive with any pathogen compared to discordant orders (OR = 3.6, 95% CI = 1.9, 6.7) (Table 1). Among the concordant orders tested, *Shigella* spp. (27% [22/81]) and *Campylobacter* spp. (12% [10/81]) were the most frequently encountered pathogens. IDP results led to a documented change in management (new prescription or tailoring of antimicrobial therapy) in 35% (28/81) of concordant cases (OR = 4.8, 95% CI = 2.1, 11.6). Physicians intended to change antimicrobial therapy in an additional five concordant cases; however, these patients were lost in follow-up. In contrast, guideline-discordant requests most frequently detected: norovirus (12% [13/112]), where half were suspected false-positives due to atypical melting curves^7^; *Clostridioides difficile* (9% [10/112]), where more than half of which tested negative for toxin by enzyme immunoassay and may have represented colonization; and EPEC (6% [7/112]), which is of questionable clinical significance (especially in adults) and not reported by our laboratory. IDP results changed antimicrobial therapy in 10% (11/112) of discordant cases, primarily treatment for *C. difficile* infection (CDI), followed by discontinuation of antibiotics in the event of a negative IDP, or initiation of treatment (one sample each with: *Shigella* spp. which failed to grow in culture, *Entamoeba histolytica* which did not confirm by enzyme immunoassay, *Giardia lamblia* which did confirm by O&P examination, and *Cryptosporidium* spp. which did not undergo further testing).

**Table 1.** IDP (Infectious diarrhea panel) results and clinical impact of guideline-concordant versus guideline-discordant orders Table 1 long description.

IDP results	Concordant orders (n = 82)(%)	Discordant orders (n = 135)(%)	Odds ratio	95% CI	*P* value
Not tested/rejected by lab^a^	1 (1)	23 (17)	0.1	0–0.4	<.01
Positive for any (≥1) target	48 (59)	38 (34)	3.6	1.9–6.7	<.01
**Bacterial:**					
* Campylobacter* spp.	10 (12)	0	∞	3.4–∞	<.01
* Clostridioides difficile* (with positive EIA^b^)	2 (2)	4 (4)	0.1	0–0.9	1.0
* Clostridioides difficile* (with negative EIA^b^)	1 (1)	6 (5)	0.2	0–1.9	.24
EAEC (Enteroaggregative *E. coli*)	3 (4)	3 (3)	1.4	0.2–10.7	.69
EPEC (Enteropathogenic *E. coli*)	4 (5)	7 (6)	0.8	0.2–3.2	.76
ETEC (Enterotoxigenic *E. coli*)	0	2 (2)	0	0–7.4	.51
* Salmonella* spp.	3 (4)	0	∞	0.6–∞	.07
* Shigella* spp.	22 (27)	3 (3)	13.4	3.8–72.7	<.01
STEC (Shiga toxin-producing *E. coli*, non-O157)	1 (1)	2 (2)	0.7	0–13.4	1.0
**Parasitic:**					
* Cryptosporidium* spp.	0	2 (2)	0	0–7.4	.51
* Entamoeba histolytica*	0	1 (1)	0	0–53.9	1.0
* Giardia* spp.	1 (1)	1 (1)	1.4	0–109.8	1.0
**Viral:**					
Adenovirus 40/41	0	1 (1)	0	0–53.9	1.0
Astrovirus	2 (2)	1 (1)	2.8	0.1–167.1	.57
Norovirus GI/GII (acceptable melting curve)	4 (5)	6 (5)	0.9	0.2–4.0	1.0
Norovirus GI/GII (atypical melting curve)	1 (1)	7 (6)	0.2	0–1.5	.14
Rotavirus	0	5 (4)	0	0–1.5	.08
Sapovirus	0	2 (2)	0	0–7.4	.51
**Clinical Impact:**					
Change in antimicrobial therapy	28 (35)	11 (10)	4.8	2.1–11.6	<.01

a Samples were manually rejected by the laboratory due to inappropriate container or labeling (n = 2), repeat testing within 7 days noticed by the medical laboratory technologist (n = 5), or admission >72 hours (n = 17).

b Samples with *C. difficile* detected by IDP were further tested by an enzyme immunoassay (EIA) for the direct detection of *C. difficile* toxin A/B and glutamate dehydrogenase antigen (C. Diff QuikChek Complete, Techlab).

Additionally, there were 10 instances in this study where laboratory protocols failed to identify samples that should have been rejected for analytical testing (repeated within seven days or inpatient admitted >72 hours).

## Discussion

Our findings demonstrate a high rate of guideline-discordant IDP ordering in the acute care setting, with 62% of overall orders not meeting clinical criteria based on BC GPAC guidelines. Similar studies have reported inappropriate testing rates exceeding 50%–60%, largely attributable to testing in patients without diarrhea, repeat requests, and use in healthcare-associated diarrhea where *C*. *difficil*e-specific testing is more appropriate.^8,9^ Our study also identified a large portion of guideline-discordant orders due to lack of diarrhea or patients where CDI was the suspected etiology.

IDP orders from inpatient wards and outpatient clinics were five times less likely to be guideline-concordant compared to ED. This may reflect front-line emergency clinicians assessing patients at the onset of acute, community-acquired illness, whereas inpatient wards may be managing patients with chronic symptoms, complicating laxative use, antimicrobial exposure, and risk factors for nosocomial CDI. Similar trends have been described by Beatty et al. (2017) and O’Neal et al. (2020), underscoring the need for targeted feedback and education across care settings.^8,9^ Guideline-concordant IDP utilization was significantly associated with the detection of bacterial pathogens (*Shigella* spp. and *Campylobacter* spp.) that have impact on patient management (especially antibiotic prescribing) and public health surveillance. These findings align with prior evaluations showing IDP provides greatest clinical value in patients with severe or invasive diarrhea when bacterial pathogens are most likely to be present.^2,4,5^ However, an important observation from this study is that results from guideline-concordant samples led to a change in management in only 35% of cases, with clinicians often noting the patient’s symptoms had already resolved or the patient could not be contacted. Conversely, guideline-discordant orders led to a change in management in 10% of cases, most often initiation of CDI treatment in patients potentially colonized with *C. difficile.* ^10^

Limitations in this study include the limited availability of clinical information from patients presenting to outpatient clinics. Potential impacts of IDP on Public Health and Infection Prevention and Control measures were not systematically evaluated; however, the focus of this diagnostic stewardship initiative was to describe clinical impact of IDP and identify opportunities for improvement. A formal cost-analysis of inappropriate IDP ordering was not performed.

This review identified numerous factors contributing to inappropriate IDP utilization, including: low adherence to clinical guidelines, orders placed by healthcare personnel who may not be familiar with test utilization criteria, and incorrect selection of *C. difficile*-specific tests. Next steps could include provider education, audit-and-feedback, electronic ordering prompts and/or automation of test exclusion, which could potentially reduce current IDP orders by up to >60%, thereby optimizing value-based healthcare.

## Figure 1. Long description

The bar graph compares the percentage of IDP orders across three locations: Emergency Department, Outpatient Clinic, and Inpatient Ward. The x-axis represents the locations, and the y-axis represents the percentage of IDP orders. The graph is a stacked bar chart with different colors representing various criteria. In the Emergency Department, the criteria are Concordant Multiple criteria met (10.3 percent), Concordant Duration greater than 7 days (5.2 percent), Concordant Severe symptoms (27.1 percent), Concordant Risk factors for severe disease (3.9 percent), Discordant No diarrhea (definition not met) (16.1 percent), Discordant Suspect CDI (2.6 percent), and Discordant Diarrhea but lack of clinical criteria above (34.8 percent). In the Outpatient Clinic, the criteria are Concordant Multiple criteria met (10.0 percent), Concordant Duration greater than 7 days (10.0 percent), Concordant Severe symptoms (30.0 percent), Concordant Risk factors for severe disease (50.0 percent). In the Inpatient Ward, the criteria are Concordant Multiple criteria met (5.8 percent), Concordant Duration greater than 7 days (1.9 percent), Concordant Severe symptoms (5.8 percent), Concordant Risk factors for severe disease (1.9 percent), Discordant No diarrhea (definition not met) (34.6 percent), Discordant Suspect CDI (19.2 percent), and Discordant Diarrhea but lack of clinical criteria above (30.8 percent). All values are approximated.

Navigate back to Figure 1..

## Table 1. Long description

The table presents a comparison of IDP (Infectious diarrhea panel) results and their clinical impact between guideline-concordant and guideline-discordant orders. It includes data on the number of orders, positive test results for various pathogens, and changes in antimicrobial therapy. The table has 27 rows and 6 columns, with headers for IDP results, concordant orders, discordant orders, odds ratio, 95% confidence interval, and P value. Notable trends include higher positivity rates for Shigella spp. and Campylobacter spp. in concordant orders, and more frequent detection of norovirus and Clostridioides difficile in discordant orders. Changes in antimicrobial therapy were more common in concordant orders.

Navigate back to Table 1..
